# A Modern French Analysis of the English Mind

**Published:** 1910-07-16

**Authors:** 


### A MODERN FRENCH ANALYSIS OF THE ENGLISH MIND.

In a recent number of " La Revue de Médecine et de Chirurgie," its distinguished editor, Dr. Helme, contributes a remarkable editorial which he entitles " Comment les français modernes regardent l'âme anglaise." The text upon which his article happens to be based is the recent translation into French of an English work on neurology.* A summary of this brilliant analysis of the English mind as judged by a French brain will doubtless be of interest to English readers, although we cannot hope to reproduce the literary elegance of the original:—

England has just lost the most noble and most universally regretted of her Kings. We in France do not understand foreign policy, since we are in the habit of regarding everything through the glasses of sentiment, nevertheless, be it said to our credit, we at once proclaimed that the death of this great prince deprived France of a true friend. A little more, and we might have called him a Frenchman, had it not been that the cause of his greatness was that he was English, nothing but English, absolutely English. Doubtless he knew and appreciated our civilisation, but had not policy led him to approach us, I believe that whilst welcoming us all warmly individually, he would not, in his capacity of King, have sought after and carried through the " entente cordiale."

When we come to know our neighbours, when we have learned to admire the immensity of human effort which has made them, for the moment, masters of the world, when we see how much they have deserved from nature, we understand how this insular race, so narrowly confined, have had to struggle more determinedly than others to gain their " place in the sun." Too often have they surpassed us by astute strategy, but we must not bear them any more grudge for that than they would have borne us had we succeeded in having all the winning cards on our side. I tell you I admire this people unreservedly. At this very moment they are passing through a formidable crisis. The future and all the traditions of the past are at stake. Yesterday the various contending parties in the battle-strife were about to cast the iron die of destiny. The king dies. A prince, as yet inexperienced, succeeds him. At once we see friends and foes, Lords and Commons, lay down their arms, and call a holy truce, to allow the newcomer on the throne time to find a solution of this most formidable of problems. This armistice may be at the cost of victory to one or other of the parties, but no matter—the future of the country and of the dynasty is at stake. If you can find in history an example of good sense, of loyalty, and of self-denial to equal this, tell it me: I know of no other.

At one of the many sumptuous receptions lately given in London by our English colleagues, receptions exemplifying the hospitality of England, so near akin to that proverbially Scottish virtue, a hospitality which has flourished more especially since the " entente cordiale," M. Huchard made a charming speech, in which he reminded his audience how William the Conqueror when he sailed for the " great island " took with him as a talisman a hair of the Apostle Peter. " This hair," said the orator, " is what has separated us for so long! "

Neither of us, be he French or English, has any cause to disown the past. If it has many a grief and anguish, it also has its glories and its heroisms. And surely it is unwise to keep in the background all that is best in the two nations, and to draw atten-

* *Le Diagnostic des Maladies Nerveuses*, par le Dr. Purves Stewart, traduit par le Professeur Scherb. Alcan, éditeur. Paris, 1910.

tion only to their points of difference. No battle-smoke ever clouded the serene atmosphere where thinkers dreamt of a mutual "entente." If we inquire of the noble shades of Harvey, of Sydenham, of Hunter, do they not bear witness to the alliance of minds, that advance-courier of the alliance of hearts? We should not forget that at the very time when our purest blood flowed in torrents on famous fields, a distinguished French scientist, Professor Roux, visited the hospitals of London, and was everywhere received with friendliness. He has left us an account of his journey in an unfamiliar book which bears the tragic date of 1815. The date alone shows conclusively that even the clash of arms, the bloody fury of battles, did not prevent the generous grasp of fraternal hands.

Ever since the eighteenth century, have not our manners, our customs, derived an inspiration from the manners and fashions of England? And during our eighteenth-century attempts to construct a constitution, did we not take as model the political organisation of our neighbours, thus attesting the admiration and attachment of our philosophers for the thoughts of those beyond the Channel? Modern writers would have it that, like Christopher Columbus, they have discovered England only in recent days. Let us read again the works of our ancestors, and we shall see how they knew and how they already appreciated the English mind.

But why this enthusiasm for England which some Frenchmen (and formerly I was one of them) have held to be excessive? The first impression which strikes the traveller in England is the complete liberty which pervades all dealings between man and man. In other countries the heavy hand of the State is felt in the smallest undertakings of life, but over there it seems concealed from every eye. The law, invisible but ever present, is to the Englishman what his goddess was to the Greek—that goddess who stood by the side of every warrior in the fray. Every citizen is so permeated by the conception of his rights and of his duties, so proud of his glorious past, so confident in his future, that the social state attains an ideal which otherwise would almost be a contradiction—the maximum protection of public authority combined with the maximum of personal liberty. And is not this special mentality even more evident at this very hour when our neighbours, calm and firm, strive to lay a hand on the Constitution itself, that magnificent edifice which they have built up throughout the centuries by their energy, their sufferings, and their genius? In other nations such a change would amount to revolution; with them everything is transformed by a simple phenomenon of evolution. This passion for liberty, conjoined with so absolute a respect for the law, could not exist save by the possession of a differentiation, a delicacy of mental processes, which is hardly to be found in any other nation.

Passing from the mass of the people to the élite, this superiority of the English race, from the psychical point of view, is still more evident. All our thinkers, from Taine onwards, were profoundly impressed by it. Need we invoke the powerful psychology of Shakespeare, psychiatrist before the term was invented, and the most admirable of psychiatrists? How could he have drawn his mighty monsters, breathed real life into Hamlet the neurasthenic, Othello the impulsive, and Lady Macbeth the hallucinated, had not the general public which applauded these Shakespearean fantasies also been able to comprehend them?

Shall I speak of Locke, the rival of our Descartes? "Since the days of Locke," says Taine, "psychology has been indigenous in England. It marches side by side with statistics and with political economy." Was not Locke the first to show that all our ideas come from sensations, and that they are graven on the "tabula rasa" of our brain by a sort on transformation of energy? These are not his exact words, but they represent his ideas in essence. To note his influence, I need but recall our own Condillac, and how he extols the English philosopher, but I feel it my duty to state that it was Locke, and after him Berkeley and Hume, who were the first inspirers of that noble gesture which burst the fetters of the lunatic, and raised these sufferers to the dignity of men and of patients. Yes, and if at the same time Pinel the Frenchman, Tuke the Englishman, Chiarugi the Tuscan, and Daquin the Savoyard, were illuminated by the same flash of light; it was because all of them were swept along in that current of ideas by forces which, starting in England, came to shake the generous heart of our own France, and by a "contre-coup," ancient Europe too.

In this hurried sketch, in which I desire to emphasise the natural adaptability, as it were, of our neighbours for psychological studies and neurological works, I must not omit the name of Darwin. When he speaks of natural selection, of the struggle for life, he is at once understood by his compatriots. Ah! is he not truly at home amongst them! On the contrary, our great Lamark, his precursor, was here disdained because he was beyond us, although he wrote his "Zoological Philosophy" in 1809 at the height of our imperialistic crisis, and at a time when the powerful oppressed the weak.

Nor do I wish to omit Spencer, to whom both nations owe so much. But if after sociology, literature, and philosophy, I turn to medicine, crowds of other witnesses will be found. Are you not struck as I am by the array of English names in neurology which figure in our nomenclature: the diseases of Little, of Parkinson, of Addison, Jacksonian epilepsy, the Argyll-Robertson sign, etc., etc.? Certainly it would be exaggeration to pretend that only the English have known how to see: but it is certain that they have so thoroughly analysed and so well described what they have seen, that history has had to attach their names to their works. And more than this. When our own Charcot aims at mastery in studying and popularising the works of others, is it not the English neuropathologists whom he takes for his models?

Since the days of the Master of the Salpêtrière the study of nervous diseases has become transformed and enlarged. In all parts of this domain a patient and thorough work of revision has been carried on. We are tracing more deeply the furrows made by our predecessors. Various types are thus modified from day to day, thanks to improved clinical methods, to micrographical discoveries, and to physiological research. Yesterday we limited ourselves to gross naked-eye lesions. To-day we are on the verge of unveiling the deepest protoplasmic secrets. Compared with French and German physicians, the English still occupy a leading place in neurology. Shall I quote among their physiologists Ferrier and Sherrington, among their clinicians, Head, Byrom Bramwell, and Mackenzie, and among their surgeons Horsley? I must pass from these and from other great names.

If the mind of our neighbours has that slowness proper to the northern races it acquires from that very fact an ardour, a precision, a tenacity, that are in a way specific. Their cold, damp climate impels them to exercise their muscles, but it condemns them also to stay indoors, to question themselves, to see nature " across man and his morals," as Taine has it. Their seafaring habit has from all time made them cultivate their faculties of observation. Condemned by the very situation of his country to cast his net around the world, the English fisherman, more than others, has had to render as perfect as possible his instruments of government—his constitution. How can one attain that almost exact foresight, that almost infallible management of human affairs, unless one has an exact intuition of the inwardness which is the essence of man?

Moreover, with the Anglo-Saxon, education is a preparation for life, instead of as with the Latin races, a thing apart from life. It is in exercising free judgment and in developing his mind as he develops the body, that the English teacher contributes his part towards the differentiation of the cerebral organ. I need go no further. To say more would be to exceed my limits.

